# Mitochondrial Biogenesis in Human Skeletal Muscle: High-Intensity Interval Training Versus Moderate-Intensity Continuous Training

**DOI:** 10.3390/ijms27219537

**Published:** 2026-10-25

**Authors:** Kevin Bischof

**Affiliations:** Department of Sport and Human Movement Science, Section for Nutrition, Exercise and Health, University of Vienna, 1150 Vienna, Austria; kevin.bischof@univie.ac.at

**Keywords:** HIIT, MICT, PGC-1α, AMPK, skeletal muscle, mitochondrial biogenesis, exercise intensity

## Abstract

Mitochondrial biogenesis is a hallmark adaptation to endurance exercise with broad implications for metabolic health and athletic performance. High-intensity interval training (HIIT) and moderate-intensity continuous training (MICT) both stimulate mitochondrial remodeling in human skeletal muscle, yet whether exercise intensity or total work performed is the principal determinant remains contested. This mini review synthesizes current evidence on the intensity-dependent activation of AMP-activated protein kinase (AMPK), p38 mitogen-activated protein kinase (p38 MAPK), and peroxisome proliferator-activated receptor gamma coactivator 1-alpha (PGC-1α)—a central transcriptional coactivator in mitochondrial biogenesis. The author critically examines the distinction between mitochondrial content and respiratory function, discusses how study design—particularly work-matched versus time-matched protocols—shapes comparative conclusions, and identifies key unresolved questions including sex-based differences, fiber-type specificity, and the role of mitochondrial dynamics. The evidence indicates that both modalities are effective but operate through overlapping yet intensity-sensitive mechanisms; the metabolic-stress hypothesis provides a testable explanatory framework. Practical implications for exercise prescription are discussed. The review further considers the coordination of mitochondrial translation and turnover, mitochondrial Ca^2+^ handling, sex-specific evidence limitations, and translational outcomes in cardiometabolic disease.

## 1. Introduction

Skeletal muscle mitochondria are central to oxidative phosphorylation, metabolic flexibility, and cellular signaling. Endurance exercise induces mitochondrial biogenesis—the coordinated transcription, translation, and assembly of mitochondrial proteins encoded by both nuclear and mitochondrial genomes—thereby supporting oxidative capacity adaptations that may contribute to metabolic health [1,2]. This adaptive response is associated with enhanced insulin sensitivity, reduced cardiometabolic risk, and improved exercise performance, making it a target of significant clinical and scientific interest. At the molecular level, exercise-induced changes in cellular energy status and contraction-associated stress engage signaling pathways involving AMP-activated protein kinase (AMPK) and p38 mitogen-activated protein kinase (p38 MAPK). These pathways contribute to the regulation of the transcriptional coactivator PGC-1α, a central mediator of mitochondrial gene expression and adaptation [2,3,4,5,6].

Two exercise training paradigms dominate contemporary endurance research. Moderate-intensity continuous training (MICT) involves sustained exercise at moderate intensity, typically below the lactate threshold, and represents the traditional cornerstone of endurance training recommendations, with well-established efficacy for improving aerobic fitness and metabolic health [7,8]. High-intensity interval training (HIIT)—comprising repeated bouts performed above the lactate threshold interspersed with passive or active recovery—generates comparable or superior cardiovascular and metabolic adaptations at substantially lower time commitments [7,9]. Sprint interval training (SIT), a subset of HIIT employing brief all-out supramaximal efforts, further compresses training volume; a single bout of all-out sprint interval exercise increased nuclear PGC-1α protein content in moderately trained individuals despite performing approximately 3.6-fold less total work than continuous exercise [10]. The demonstration that such low training volumes can elicit robust metabolic adaptations comparable to traditional endurance training has reshaped exercise recommendations and generated considerable scientific debate [7,11].

A fundamental mechanistic question remains: does exercise intensity, independent of total mechanical work, drive qualitatively or quantitatively distinct mitochondrial adaptations? Resolution of this question carries important practical implications. For time-constrained adults, HIIT may provide a time-efficient option; in clinical or older populations, modality choice should instead account for individual capacity, tolerability and clinical assessment. This review addresses the question by examining intensity-dependent molecular signaling, the content-versus-function distinction in mitochondrial outcomes, and the methodological variables that complicate direct comparisons between training modalities.

### Review Scope and Literature Identification

**Figure 1:**
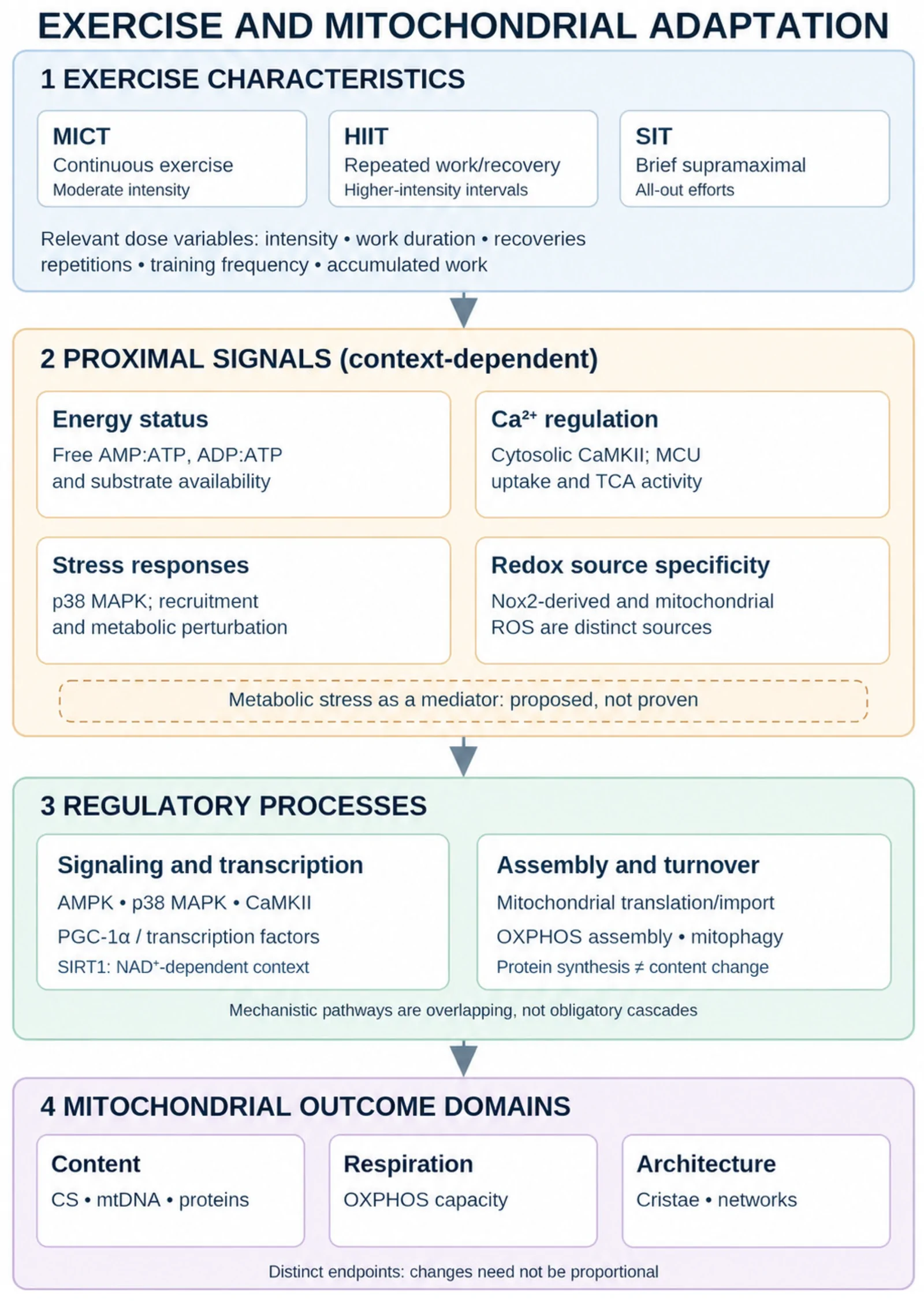
Conceptual model of exercise-related mitochondrial remodeling in skeletal muscle (not to scale). Exercise-modality characteristics and dose influence overlapping energetic, Ca^2+^-dependent, and redox-associated signals that can converge on mitochondrial transcription, protein synthesis/assembly, and quality control. The dashed metabolic-stress box denotes a proposed, incompletely validated mediator rather than a necessary causal pathway [5,12,13,14]. The human evidence principally concerns acute kinase/PGC-1α responses, whereas a causal contribution of Nox2 to selected HIIT adaptations derives from genetically modified mice [15]. Mitochondrial content, respiratory function, and architecture are distinct and potentially dissociable outcomes [16,17,18,19,20,21,22].

This narrative mini-review uses citation-led identification of relevant mechanistic and comparative exercise studies rather than a prospectively registered, comprehensive database strategy. Additional focused searches of PubMed-indexed records and publisher websites were undertaken in August-September 2026, using combinations of terms including but not restricted to “HIIT”, “MICT”, “skeletal muscle”, “mitochondrial translation”, “mitophagy”, “mTORC1”, “mitochondrial calcium”, “sex hormones”, “menstrual cycle”, “metabolic syndrome”, “type 2 diabetes” and “older adults”; reference lists of pertinent reviews were also examined. Human intervention studies with skeletal-muscle molecular or functional outcomes were prioritized for comparative conclusions, whereas mechanistic cell and rodent studies were used to contextualize candidate pathways and identified explicitly by model (Figure 1).

## 2. Molecular Signaling: Converging but Intensity-Sensitive Pathways

### 2.1. AMPK Activation and Energy Sensing

AMPK directly phosphorylates PGC-1α and contributes to the regulation of mitochondrial gene expression [3]. Separately, experimental evidence from skeletal muscle cells and mice indicates that AMPK-dependent changes in NAD^+^ availability can modulate SIRT1-associated PGC-1α deacetylation [23]. However, exercise-induced PGC-1α deacetylation and mitochondrial biogenesis have also been observed in mice lacking skeletal-muscle SIRT1 deacetylase activity [24]. Consequently, the involvement of SIRT1 in exercise-induced mitochondrial adaptation appears context-dependent, and differential regulation following HIIT versus MICT in humans remains unestablished.

Mechanistically, AMP, ADP, and ATP compete for exchangeable nucleotide-binding sites on the regulatory AMPK γ-subunit. AMP promotes allosteric activation, enhances upstream kinase-dependent phosphorylation of Thr172 in the catalytic α-subunit, and protects against Thr172 dephosphorylation, whereas ADP primarily inhibits dephosphorylation; the relative sensitivities to these nucleotides vary among γ-subunit isoforms [25,26]. During skeletal-muscle contraction, increases in estimated free AMP:ATP and ADP:ATP ratios can occur without an immediate reduction in total muscle ATP, although substantial ATP depletion may occur during prolonged or exhaustive high-intensity exercise [6,17,27].

Work-matched interval and continuous running in trained men elicited comparable AMPK (Thr172) and p38 MAPK phosphorylation immediately after exercise, alongside similar PGC-1α mRNA responses 3h later [12]. Duration-matched interval and continuous all-out sprint cycling in trained individuals likewise produced comparable AMPK phosphorylation and PGC-1α mRNA responses [13] (a list of human studies investigating metabolic and molecular outcomes is illustrated in Table 1). Thus, changing the distribution of exercise work does not necessarily produce greater acute signaling when relevant features of exercise exposure are matched; neither comparison establishes metabolic stress as sole causal determinant. These studies predominantly involved men and do not establish equivalent responses across sexes or ovarian-hormone states [28,29,30,31].

Animal models, however, reveal tissue-specific nuances. Six weeks of HIIT upregulated AMPKα1 gene expression and mitochondrial transcripts (Cs, Nd1, Cox2) preferentially in oxidative soleus muscle compared with glycolytic extensor digitorum longus, whereas MICT had negligible transcriptional effects in either muscle type; notably, AMPK protein phosphorylation (p-AMPKα/AMPKα ratio) was not significantly altered by either training protocol [34]. Separately, HIIT promoted markers of mitochondrial biogenesis in gastrocnemius of elderly female rats more effectively than MICT [35]. Whether these rodent findings reflect genuine intensity-driven effects translatable to human skeletal muscle warrants systematic investigation with fiber-type resolution.

AMPK additionally facilitates TFAM expression through downstream transcriptional cascades, coupling energy sensing to the replication and transcription of mitochondrial DNA [2]. The magnitude and transcriptional profile of the exercise-induced AMPK–PGC-1α response vary between individuals and are influenced by training history. In human skeletal muscle, trained and untrained participants show distinct acute molecular responses to exercise, while short-term training can alter the subsequent PGC-1α and mitochondrial-protein response [36,37]. Genetic evidence is more specific: the PPARGC1A Gly482Ser gene variant has been associated with impaired exercise-induced slow-twitch muscle-fiber transformation, although carriers can still increase mitochondrial density and oxidative-enzyme activity; therefore, this variant should not be described as preventing mitochondrial biogenesis [38]. Evidence that PPARA polymorphisms or baseline mitochondrial density directly determine the acute AMPK–PGC-1α response to standardized exercise remains insufficient, so these factors should not be presented as established moderators without more specific supporting studies. This heterogeneity and uncertainty have important implications for the design and interpretation of HIIT versus MICT comparisons in small-sample studies.

### 2.2. p38 MAPK and the Metabolic Stress Hypothesis

The p38 mitogen-activated protein kinase pathway responds to contractile activity and the associated metabolic perturbations of exercise, and phosphorylates PGC-1α, contributing to the transcriptional induction of mitochondrial genes [2,4]. Calcium-dependent signaling provides a further intensity-sensitive input: higher-intensity exercise is associated with greater activation of Ca^2+^/calmodulin-dependent protein kinase II (CaMKII) and AMPK, alongside greater exercise-induced PGC-1α expression [5]. Ca^2+^-sensitive signaling pathways, including CaMKII and p38 MAPK, can therefore contribute to the regulation of PGC-1α and mitochondrial gene expression in parallel with energy-sensitive AMPK signaling [2,6]. Experimental evidence indicates that p38 MAPK can regulate PGC-1α through distinct post-translational and transcriptional mechanisms. Direct p38-dependent phosphorylation can enhance PGC-1α protein stability and coactivator activity, whereas downstream ATF2 signaling and MEF2-dependent promoter regulation contribute to contraction-induced PGC-1α gene expression [4,6,39,40,41,42]. Although these mechanisms have been demonstrated primarily in cellular and rodent models, their differential contribution to mitochondrial adaptations following HIIT versus MICT in human skeletal muscle remains to be established.

The metabolic-stress hypothesis proposes that exercise-induced metabolic perturbations may contribute to mitochondrial biogenesis-related signaling beyond nominal intensity or total mechanical work [14]. Candidate influences include changes in free adenine nucleotides, muscle lactate, pH, glycogen availability, and redox state; these correlated variables have not been established as independently necessary or sufficient mediators in human HIIT. A proposed metabolic-stress “sweet spot” remains a hypothesis rather than validated prescription threshold [14,43].

Reactive oxygen species may regulate contraction-associated signaling through anatomically distinct sources. In genetically modified mice lacking Nox2, selected HIIT adaptations involving oxidative-metabolism-related protein markers and mitochondrial-network remodeling were attenuated [15]. This loss-of-function finding should not be interpreted as direct evidence of a Nox2 requirement in human HIIT or equated with mitochondria-derived ROS; compartment-specific mechanisms are examined below in Section 2.2.2.

#### 2.2.1. Evidence for and Against the Metabolic-Stress Hypothesis

Supporting human evidence includes the work-matched supramaximal exercise protocols investigated by Fiorenza et al. [14], in which greater intramuscular metabolic perturbations were associated with enhanced CaMKII and p38 MAPK phosphorylation and mitochondrial biogenesis-related transcriptional responses. Furthermore, sprint-interval exercise increased nuclear PGC-1α protein abundance compared with continuous exercise despite substantially lower total work [10], while a separate acute sprint-interval study demonstrated increased nuclear PGC-1α abundance without a continuous-exercise comparator [44]. However, these findings establish associations and protocol-specific responsiveness rather than direct causation. Exercise intensity, motor-unit recruitment, Ca^2+^ signaling, catecholamine responses, and other contraction-associated signals may vary alongside metabolic perturbations, preventing the independent contributions of lactate accumulation, acidification, or glycogen depletion from being established [5,10,14].

Several findings challenge a simple relationship between metabolic stress and mitochondrial signaling. Work-matched interval and continuous running elicited comparable AMPK and p38 MAPK phosphorylation and PGC-1α mRNA responses despite greater blood lactate accumulation following interval exercise [12]. Similarly, duration-matched interval and continuous all-out sprint cycling produced comparable AMPK and PGC-1α responses despite differences in total work [13]. Fiorenza et al. [14] also observed similar PGC-1α mRNA responses following speed-endurance and higher-volume continuous exercise despite different metabolic profiles. Chronic adaptations likewise vary across protocols: two weeks of work-matched interval training produced greater increases in citrate synthase activity and mass-specific mitochondrial respiration than continuous training [19], whereas a four-week comparison matched for absolute and mean intensity, exercise duration, and total work increased several mitochondrial content markers following continuous but not intermittent training [32]. Importantly, the latter study isolated exercise pattern rather than intensity, and neither chronic comparison directly established differences in intramuscular metabolic stress. Collectively, these findings suggest that metabolic stress may contribute to exercise-induced mitochondrial signaling but is neither an established exclusive mediator nor a sufficient predictor of long-term mitochondrial adaptation.

Validation of the metabolic-stress hypothesis requires prespecified, testable predictions concerning individual metabolic mediators and downstream mitochondrial outcomes. Experimental manipulation of carbohydrate availability provides one approach: reduced muscle glycogen availability has been associated with enhanced exercise-induced p53 signaling [45], whereas graded reductions in pre-exercise glycogen concentration did not augment nuclear AMPK protein abundance or PGC-1α mRNA responses during standardized HIIT [46]. These divergent findings highlight the importance of the specific metabolic variable, signaling endpoint, and exercise protocol examined. If exercise protocols producing comparable intramuscular metabolic perturbations consistently elicited divergent signaling and chronic adaptations, this would challenge the proposition that metabolic stress fully mediates intensity-dependent responses. Future studies should combine controlled crossover exercise protocols with repeated muscle sampling, direct assessment of intramuscular energetic and metabolic variables, and longitudinal measurements of mitochondrial protein synthesis, content, respiratory function, and turnover. Complementary mechanistic experiments targeting individual metabolic or contraction-associated pathways are needed to establish causality rather than infer it from associations alone.

#### 2.2.2. Mitochondrial Ca^2+^ Homeostasis and Compartment-Specific Redox Signaling

Beyond cytosolic Ca^2+^/CaMKII signaling, mitochondrial Ca^2+^ uptake represents an additional mechanism linking muscle contraction to oxidative metabolism. During excitation–contraction coupling, Ca^2+^ release from the sarcoplasmic reticulum (SR) generates local Ca^2+^ microdomains that facilitate mitochondrial uptake through the mitochondrial calcium uniporter (MCU), particularly in mitochondria positioned near SR Ca^2+^-release structures [22,47]. Within physiological limits, increases in mitochondrial matrix Ca^2+^ stimulate oxidative metabolism by directly activating isocitrate and α-ketoglutarate dehydrogenases and indirectly promoting pyruvate dehydrogenase activation through Ca^2+^-sensitive pyruvate dehydrogenase phosphatase. These mechanisms enhance the supply of reducing equivalents to the respiratory chain and support ATP production [22]. Experimental evidence from skeletal-muscle-specific MCU-deficient mice further demonstrates that impaired mitochondrial Ca^2+^ uptake alters glucose oxidation, substrate preference, muscle function, and exercise performance [48]. Conversely, excessive or sustained mitochondrial Ca^2+^ accumulation can compromise organelle homeostasis. Thus, maintaining mitochondrial Ca^2+^ within physiological limits is important for skeletal-muscle function. However, whether specific HIIT protocols cause excessive mitochondrial Ca^2+^ accumulation, or whether differences in mitochondrial Ca^2+^ handling contribute to divergent adaptations following HIIT and MICT in humans, remains unknown [22].

Mitochondrial Ca^2+^-dependent metabolic regulation should also be distinguished from compartment-specific redox signaling. Nox2-derived ROS and mitochondria-derived ROS originate from distinct subcellular sources and may exert different signaling effects. Experimental evidence from genetically modified mice indicates that Nox2 activity is required for several skeletal-muscle adaptations to HIIT, including mitochondrial-network remodeling and changes in oxidative-metabolism-related proteins [15]. Although SR Ca^2+^ release, mitochondrial uptake, activation of Ca^2+^-sensitive dehydrogenases, and changes in NADH availability provide a plausible mechanism linking contractile activity to mitochondrial bioenergetics, this sequence does not necessarily imply increased mitochondrial ROS production or obligatory downstream activation of AMPK/PGC-1α signaling during human HIIT. Furthermore, subsarcolemmal and intermyofibrillar mitochondrial populations differ in their anatomical organization, while intermyofibrillar mitochondria are positioned near SR Ca^2+^-release structures [47,49,50]. Human morphological evidence also demonstrates fiber-type-dependent mitochondrial organization [51]. Whether these structural characteristics contribute to fiber-type-specific mitochondrial Ca^2+^ signaling and differential adaptations to HIIT versus MICT remains to be established.

### 2.3. PGC-1α: Isoforms, Nuclear Translocation, and Transcriptional Targets

PGC-1α occupies the apex of the transcriptional hierarchy governing mitochondrial biogenesis. Acting as a transcriptional coactivator, PGC-1α interacts with nuclear respiratory factors (NRF-1 and NRF-2), estrogen-related receptors (particularly ERRα), and other transcription factors to coordinate a broad transcriptional program of nuclear-encoded mitochondrial genes. Through activation of NRF-1/2, PGC-1α also promotes expression of mitochondrial transcription factor A (TFAM), which is required for the transcription and replication of mitochondrial DNA [4,52]. Alternative promoter usage and splicing of PPARGC1A generate PGC-1α variants with distinct structural and functional properties. Canonical PGC-1α1 is primarily associated with mitochondrial biogenesis and oxidative gene expression, whereas alternative isoforms PGC-1α2 and PGC-1α3 have been implicated in distinct transcriptional and RNA-processing programs, although their physiological roles in human exercise adaptation remain incompletely understood [53,54,55]. In contrast, the truncated PGC-1α4 isoform has been linked to increased IGF1 expression, myostatin suppression, and skeletal-muscle hypertrophy in cellular and rodent models [54]. Human studies demonstrate that alternative-promoter-derived PGC-1α-b protein increases rapidly following endurance exercise [56], whereas transcripts encoding truncated PGC-1α variants can be induced by both endurance and resistance exercise [57]. These findings indicate that exercise modalities may differentially regulate PGC-1α isoform expression, but whether individual isoforms causally mediate distinct mitochondrial content or respiratory adaptations following HIIT versus MICT remains unresolved.

Nuclear accumulation of PGC-1α, which facilitates its transcriptional coactivator function, can increase acutely following selected high-intensity exercise protocols. A single bout of low-volume HIIT (4 × 30 s all-out cycling with 4 min recovery) increased nuclear PGC-1α abundance within 3 h in healthy recreationally active men [44]. Similarly, sprint-interval exercise (4 × 30 s all-out cycling sprints) increased nuclear PGC-1α protein content immediately after exercise, whereas 24 min of continuous cycling at 63% of peak power did not [10]. However, work-matched comparisons report similar PGC-1α mRNA responses between interval and continuous exercise [12], and duration-matched comparisons yield similar findings [13]. Together, these findings suggest that any apparent intensity-related advantage for PGC-1α signaling may depend on the experimental protocol, participant characteristics, and the specific molecular outcome assessed, particularly nuclear protein abundance versus mRNA expression.

PGC-1α-driven transcription regulates the expression of genes involved in oxidative phosphorylation, the TCA cycle, fatty acid oxidation, and antioxidant defense, thereby contributing to the expansion of skeletal muscle oxidative capacity [58,59]. Recent phosphoproteomic analyses have revealed exercise-intensity-specific kinase, substrate, and signaling networks extending beyond canonical AMPK and p38 MAPK pathways [60]. These findings suggest that additional signaling mechanisms may contribute to the regulation of mitochondrial adaptations, although their functional links to PGC-1α and mitochondrial biogenesis remain to be established.

Beyond phosphorylation and deacetylation, PGC-1α is subject to additional post-translational regulatory mechanisms. PRMT1-mediated arginine methylation has been shown to enhance its transcriptional coactivator activity, whereas SCF–Fbw7-dependent ubiquitination promotes proteasomal degradation, thereby regulating PGC-1α protein stability and transcriptional availability. Furthermore, GCN5-mediated acetylation suppresses PGC-1α coactivator activity [39,61]. Much of the site-specific evidence derives from biochemical systems and non-exercise or nonhuman models, so these mechanisms are candidate explanatory axes rather than demonstrated differences between HIIT and MICT in human skeletal muscle.

### 2.4. Mitochondrial Translation, Proteostasis, and Organelle Turnover

Mitochondrial biogenesis requires the coordinated expression of nuclear- and mitochondrial-encoded proteins, their translation, import, and assembly into functional oxidative phosphorylation (OXPHOS) complexes [2]. Whereas most mitochondrial proteins are encoded by the nuclear genome, synthesized by cytosolic ribosomes, and subsequently imported into mitochondria, the mammalian mitochondrial genome encodes 13 polypeptides that are synthesized by mitochondrial ribosomes. Mitochondrial translation involves distinct initiation, elongation, and termination mechanisms, followed by the insertion of nascent polypeptides into the inner mitochondrial membrane and their coordinated assembly with nuclear-encoded OXPHOS subunits [62,63]. Within this regulatory framework, TFAM contributes to mtDNA maintenance, transcription, and replication, whereas mitoribosomal translation and respiratory-complex assembly depend on additional regulatory machinery [2,62]. Consequently, increases in TFAM or PPARGC1A expression alone are insufficient to establish increased mitochondrial protein synthesis or the accumulation of fully assembled, functional respiratory complexes.

Cytosolic translational control provides another regulatory layer in exercise-induced mitochondrial adaptation. Mechanistic target of rapamycin complex 1 (mTORC1) regulates translation initiation through phosphorylation of downstream effectors, including 4E-BP1 and S6K1, whereas AMPK activation under energetic stress can suppress mTORC1 signaling [6,64]. However, this regulatory relationship is not strictly antagonistic, as concurrent activation of AMPK- and mTORC1-associated signaling has been observed following endurance exercise in humans [64]. Furthermore, experimental evidence from mice indicates that mitochondrial-fraction protein synthesis can be maintained despite chronic rapamycin-mediated inhibition of mTORC1 signaling and reductions in cytosolic protein synthesis [65]. Conversely, mTOR inhibition has also been shown to impair mitochondrial gene expression and respiratory function through mechanisms involving the YY1–PGC-1α transcriptional complex [66]. Collectively, these findings indicate that the relationship between AMPK–mTORC1 signaling and mitochondrial adaptation is context-dependent and cannot be reduced to a simple antagonistic model. Whether differences in this signaling axis contribute to divergent mitochondrial adaptations following HIIT and MICT in human skeletal muscle remains unresolved and requires direct assessments of mitochondrial protein synthesis, protein import, and respiratory-complex assembly.

Net mitochondrial content reflects the balance between mitochondrial biogenesis and degradation, with selective mitochondrial clearance through mitophagy contributing to organelle quality control [67]. Mechanistically, PINK1 accumulation on dysfunctional mitochondria can promote Parkin recruitment and ubiquitination of outer mitochondrial membrane proteins, thereby facilitating the recruitment of autophagic machinery. In parallel, mitochondrial receptors including BNIP3, NIX/BNIP3L, and FUNDC1 can interact with LC3-associated autophagic machinery through receptor-mediated mechanisms that do not necessarily require Parkin [67]. Experimental evidence from mice further supports the involvement of Parkin and AMPK–ULK1 signaling in exercise-induced mitophagy [68,69]. However, changes in the abundance of mitophagy-associated proteins, including LC3, p62, PINK1, and Parkin, do not independently establish mitophagic flux, as they cannot reliably distinguish increased mitochondrial degradation from altered autophagosome formation or impaired lysosomal clearance [70,71]. Accordingly, changes in mitochondrial content cannot be attributed exclusively to enhanced biogenesis without considering concurrent mitochondrial turnover. Direct measurements of mitochondrial synthesis and degradation, particularly in work-matched comparisons of HIIT and MICT in human skeletal muscle, are needed to establish whether exercise intensity differentially regulates these processes.

## 3. Mitochondrial Content Versus Respiratory Function

A conceptually important but frequently conflated distinction separates mitochondrial content from mitochondrial function. Mitochondrial content is commonly estimated using indirect markers such as citrate synthase (CS) activity, mitochondrial DNA (mtDNA) copy number, or mitochondrial membrane proteins such as VDAC/porin, whereas mitochondrial function is typically assessed by high-resolution respirometry of permeabilized muscle fibers or isolated mitochondria, quantifying ADP-stimulated and/or maximal respiratory capacity with complex I-, complex II-, or convergent complex I+II-supported substrates. These two dimensions can dissociate: increases in mitochondrial content do not necessarily translate proportionally into greater respiratory capacity, while improvements in mitochondrial respiratory function can occur without corresponding increases in conventional markers of mitochondrial content [16,17,18]. MacInnis et al. provided evidence that exercise intensity can modulate mitochondrial adaptations in a two-week single-leg cycling study [19]. Interval cycling matched for total work and session duration to continuous cycling produced greater increases in CS activity and mass-specific mitochondrial oxidative phosphorylation capacity, including complex I- and complex I+II-linked respiration [19]. However, respiration normalized to CS activity was not different between conditions, suggesting that the superior respiratory capacity following interval training was largely accompanied by greater mitochondrial content rather than enhanced respiratory function per unit of mitochondrial content. In contrast, two weeks of HIIT in previously sedentary adults increased mitochondrial respiration per unit of mitochondrial protein across lipid, complex I-, complex I+II-, and complex II-supported substrates without corresponding increases in whole-tissue mitochondrial respiratory-chain protein abundance [20]. These findings suggest that early HIIT adaptations may involve complex-specific remodeling of respiratory function without proportional changes in conventional content markers [20].

The importance of cristae architecture—the inner membrane invaginations that house the respiratory machinery—has emerged as a key determinant of mitochondrial oxidative capacity. HIIT increased mitochondrial cristae density in skeletal muscle of men with type 2 diabetes [21], indicating that mitochondrial remodeling can extend beyond changes in mitochondrial volume and abundance. In parallel, training intensity modulated mitochondrial respiration and PGC-1α protein content in healthy men, whereas conventional markers of mitochondrial content, including CS activity and mitochondrial respiratory-chain protein abundance, remained unchanged across training conditions [33]. This pattern—training-intensity-dependent increases in mitochondrial respiratory function occurring without corresponding changes in conventional content markers—underscores the limitations of relying solely on mitochondrial content proxies.

The time course of mitochondrial remodeling also influences interpretation. Short HIIT interventions may reveal changes in respiration before corresponding increases in conventional mitochondrial content markers become detectable [20]. For example, four weeks of SIT increased mitochondrial respiration and PGC-1α protein content while CS activity and several other conventional content markers remained unchanged [33]. A recent systematic review and meta-analysis indicates that MICT can increase mitochondrial volume density and CS activity, although the findings vary with training duration and other methodological characteristics [8]. Consequently, apparent differences between HIIT and MICT may depend on intervention duration and whether content or respiratory function is evaluated.

The practical implication is that studies relying solely on conventional mitochondrial content markers may fail to detect differences in functional adaptation between training modalities. Future work combining parallel assessment of mitochondrial content and respiratory function from the same muscle biopsy—ideally with fiber-type resolution—is needed to better characterize the qualitative and quantitative contributions of HIIT and MICT to mitochondrial adaptation.

## 4. Methodological Considerations: Study Design as a Confounding Variable

Perhaps the most consequential source of discordance in the HIIT versus MICT literature is the experimental matching strategy. Two principal designs exist, each answering a different question:

1. Work-matched designs equate the total mechanical work performed in each condition, thereby controlling one important dimension of exercise dose, although recovery structure and other protocol features may still differ. Under this constraint, work-matched interval and continuous running produced similar acute increases in PGC-1α mRNA and AMPK and p38 phosphorylation in human skeletal muscle [12]. However, longer-term adaptations do not necessarily follow the same pattern. In a 4-week single-leg cycling study in which absolute intensity, total work, training time, and volume were matched, continuous training increased several markers of mitochondrial content, including CS activity, mitochondrial protein content, and subsarcolemmal mitochondrial volume, whereas intermittent training did not [32]. Conversely, in an earlier 2-week single-leg cycling study matched for total work and session duration, HIIT produced greater increases in CS activity and mass-specific mitochondrial respiratory capacity than MICT [19]. Together, these findings indicate that matching total work does not eliminate differences between training modalities and that the apparent effect of intensity depends strongly on the mitochondrial endpoint assessed.
2. Time-matched designs equate session duration, whereas unmatched comparisons evaluate protocols as prescribed; either approach can involve substantial differences in accumulated work. Under these conditions, low-volume SIT can produce mitochondrial and metabolic adaptations comparable to those achieved with traditional endurance training despite markedly lower training volume and time commitment [11]. Other studies demonstrate increases in mitochondrial protein content and oxidative capacity following very low-volume HIIT interventions, although these studies did not include a MICT comparator [72,73]. Collectively, these findings support the time-efficiency of high-intensity training for inducing mitochondrial adaptations. The CrossTalk debate in The Journal of Physiology [74] encapsulates this tension: advocates for intensity argue that high-intensity exercise provides a potent stimulus for mitochondrial adaptation, whereas proponents of volume emphasize the importance of cumulative training exposure. Both perspectives find empirical support, in part because the relative contribution of intensity and volume depends on the experimental design and the mitochondrial endpoint assessed.

Within HIIT, exercise dose is determined by multiple interacting variables, including interval intensity, work-bout duration, repetition number, recovery duration and intensity, training frequency, and accumulated exercise volume [7,75]. Consequently, low-volume and higher-volume interval protocols, as well as supramaximal SIT and submaximal HIIT, should not be considered physiologically interchangeable, even when classified under the broader category of interval training [7,11,72,73]. Comparisons that systematically control total work, exercise duration, and recovery characteristics are needed to isolate the contributions of individual prescription variables. Indeed, work-matched high-intensity exercise protocols can elicit different degrees of metabolic perturbation and mitochondrial biogenesis-related molecular signaling, indicating that total work alone does not fully characterize the exercise stimulus [14]. Accordingly, greater peak intensity should not be assumed to produce proportionally greater mitochondrial adaptations, particularly when total work and accumulated exercise exposure differ [7,75,76].

Other methodological influences include baseline training status, the use of absolute versus relative intensity targets, and the timing of muscle biopsies relative to exercise. Fiber-type composition is another important consideration because mitochondrial content and oxidative capacity differ substantially between fiber types; however, mitochondrial adaptations are often assessed at the whole-muscle level rather than being resolved according to fiber type [49,50,76]. Because foundational acute biopsy studies have predominantly recruited men [5,10,12,13,14,44,60], inferred modality effects may be sex-specific or underpowered to detect hormonal-state interactions. Future protocols should predefine menstrual-phase verification, hormonal contraceptive exposure, menopause/sex-hormone status and relevant medications while avoiding post hoc assumptions of fixed phase effects [28,29,30,31].

Inter-individual variability in training responses represents an additional source of heterogeneity that may complicate comparisons between HIIT and MICT, particularly in small-sample studies. Moreover, observed between-participant differences in training responses may reflect a combination of true biological heterogeneity, measurement error, and within-participant variability rather than stable differences in “trainability” [77,78]. This is particularly relevant when comparing mitochondrial adaptations across small studies, where substantial between-participant variability can reduce statistical precision and obscure modality-specific effects. Designs that reduce between-participant variability, such as within-subject comparisons where appropriate, together with rigorous standardization of exercise dose and biopsy timing, may therefore improve the interpretability of HIIT versus MICT comparisons.

## 5. Open Questions and Future Directions

*Sex-based differences*. The overwhelming majority of mechanistic studies of interval training have been conducted in males, and evidence regarding skeletal muscle mitochondrial responses to HIIT in females remains limited and conflicting [28]. Emerging studies suggest that the magnitude of some mitochondrial adaptations may differ between sexes. For example, a tendency for greater mitochondrial protein synthesis has been reported in men than women following low-volume SIT [79], while mitochondrial respiration increased in older men but not women following six weeks of HIIT [80]. The mechanistic basis of these observations remains unclear, although sex-related differences in substrate metabolism and hormonal factors may contribute. Adequately powered studies directly comparing males and females, with careful consideration and reporting of menstrual cycle phase, menopausal status, and hormonal contraceptive use, are therefore warranted.

Estrogen receptor-α (ERα) signaling has been implicated in skeletal-muscle mitochondrial homeostasis and insulin action. Constitutive muscle-specific ERα deletion in mice has been associated with impaired oxidative metabolism, altered mitochondrial quality control, and reduced insulin sensitivity [29,81]. However, these findings are not uniform across experimental models, as inducible ERα deletion in adult female mice did not impair skeletal-muscle mitochondrial function or glucose uptake [82]. Thus, the contribution of ERα signaling appears model-dependent, and its role in differential mitochondrial adaptation following HIIT versus MICT in women remains to be investigated. Furthermore, estradiol and progesterone can influence exercise substrate utilization, antioxidant regulation, and intracellular signaling pathways, including Ca^2+^- and AMPK-associated mechanisms [31]. Human experimental evidence also indicates that estradiol exposure can modify exercise-induced PGC-1α mRNA responses, although consistent menstrual-phase-dependent differences have not been established [83]. These observations suggest that ovarian hormone status may influence the metabolic and molecular environment in which exercise-induced mitochondrial adaptations occur. However, whether these mechanisms translate into differential AMPK/PGC-1α activation or mitochondrial adaptations following HIIT versus MICT in women remains to be determined.

The early follicular phase, characterized by relatively low estradiol and progesterone concentrations; the late follicular phase, characterized by elevated estradiol and low progesterone; and the mid-luteal phase, characterized by elevated progesterone alongside moderately elevated estradiol, represent distinct endocrine environments that may influence exercise metabolism [31]. Combined hormonal contraceptives introduce exogenous estrogenic and progestogenic compounds and generally suppress ovulation and endogenous ovarian hormone fluctuations, although their endocrine effects depend on the formulation and administration regimen [84]. Studies seeking to determine menstrual-phase-specific physiological or molecular responses should therefore verify hormonal status using appropriate biochemical assessments, ideally alongside menstrual-cycle tracking and ovulation testing, rather than relying exclusively on calendar-based phase classification [84]. Importantly, a systematic review and meta-analysis reported only trivial average differences in exercise performance across menstrual-cycle phases, with substantial between-study heterogeneity and predominantly low-quality evidence [30]. However, these performance-related findings cannot be interpreted as direct evidence for the presence or absence of menstrual-phase-dependent differences in exercise-induced mitochondrial signaling or adaptations in human skeletal muscle.

*Fiber-type specificity*. HIIT progressively recruits a greater proportion of type II fibers, which generally have lower oxidative capacity than type I fibers and may therefore experience a greater metabolic perturbation during high-intensity exercise. However, evidence for preferential mitochondrial adaptation of type II fibers following HIIT or SIT remains limited and equivocal. In a 12-week fiber-type-resolved comparison of MICT and SIT, mitochondrial content markers increased similarly in type IIa fibers, whereas increases in type I fiber-specific mitochondrial markers were observed following MICT but not SIT [85]. Thus, these findings do not support the notion that the mitochondrial effects of SIT are primarily driven by disproportionate adaptation of type II fibers. Similarly, continuous and interval exercise elicited comparable increases in PGC-1α and PDK4 mRNA in both type I and type II fibers in untrained individuals [86], indicating that greater type II fiber recruitment does not necessarily translate into a larger transcriptional response in these canonical markers. Whole-muscle homogenates may nevertheless obscure fiber-type-specific adaptations, particularly when responses differ between fiber types or when changes occur selectively within specific fiber populations. Whether mitochondrial adaptations are accompanied by longer-term changes in fiber-type phenotype and myosin heavy-chain composition remains an important question for future research. Fiber-type-resolved approaches, including single-fiber analyses, immunofluorescence, and fiber-specific proteomics, will therefore be valuable for determining how exercise intensity shapes mitochondrial remodeling across the heterogeneous skeletal muscle fiber pool.

*Mitochondrial dynamics*. Fission (DRP1, FIS1) and fusion (MFN1/2, OPA1) processes contribute to mitochondrial morphology, quality control, and functional integration. Emerging evidence suggests that HIIT and MICT can induce distinct patterns of mitochondrial network remodeling. In a recent study in young men, both HIIT and MICT altered mitochondrial network morphology after six weeks, with MICT producing a more grid-like arrangement and HIIT promoting a more strongly longitudinally oriented network; HIIT also produced more pronounced changes in several mitochondrial fusion- and fission-related transcripts [87]. However, the functional significance of these distinct morphological adaptations remains unclear, as respiratory function was not comprehensively assessed in relation to network architecture [87]. In oxidative rodent muscle, HIIT increased expression of several mitochondrial dynamics-related transcripts, including Mfn2, Opa1, and Drp1 relative to untrained animals, while Fis1 expression was higher than with MICT; these effects were observed in soleus but not fast-twitch EDL muscle [34]. Evidence regarding exercise-induced mitophagy is even more limited. In diet-induced obese mice, prolonged HIIT altered markers of mitochondrial quality control, including Drp1 phosphorylation and the p62/LC3B autophagy machinery, but the study did not directly assess mitophagic flux or compare HIIT with MICT [70]. Controlled human studies combining measurements of mitochondrial morphology, dynamics, respiratory function, and mitophagic flux, ideally with fiber-type resolution, are therefore needed to determine whether exercise intensity produces distinct adaptations in mitochondrial quality control. Importantly, observations of fusion/fission proteins or autophagy-associated markers do not establish mitochondrial turnover rates; direct, work-matched human HIIT-MICT comparisons of mitophagic flux remain needed [67,88].

*Transcriptomic and phosphoproteomic resolution*. Unbiased transcriptomic profiling can reveal exercise-modality-specific skeletal muscle gene expression signatures extending beyond the candidate pathways traditionally emphasized in exercise biology. Recent RNA-sequencing data from murine skeletal muscle identified distinct transcriptional adaptations following HIIT versus MICT, including differential regulation of FoxO, MAPK, and PI3K-AKT signaling pathways [89]. Although these findings require validation in human skeletal muscle, they illustrate the potential of transcriptome-wide approaches to identify molecular responses not captured by candidate-gene analyses. Phosphoproteomic analyses in humans have likewise identified exercise-intensity-specific signaling networks involving distinct kinases, substrates, and phosphosites, including candidate regulatory events that extend beyond canonical exercise-signaling pathways [60]. These data provide candidate mechanistic entry points that warrant functional validation. Multi-omic integration of transcriptional networks, phosphoproteomic signaling maps, and concurrent high-resolution respirometry in the same individuals could help identify molecular signatures most strongly associated with functional, rather than solely content-based, mitochondrial adaptation.

*Clinical translation*. While HIIT is increasingly investigated as an exercise strategy in cardiometabolic disease, the optimal dose, format, feasibility, and safety for maximizing skeletal-muscle mitochondrial adaptations in insulin-resistant, obese, and older populations remain incompletely defined. Whether the intensity requirements and molecular signaling responses observed in healthy adults are directly transferable to clinical populations remains unclear. Studies directly comparing clinically tolerable interval formats with continuous training are needed.

The clinical relevance of skeletal-muscle mitochondrial adaptations is best established by linking mitochondrial remodeling to functional metabolic outcomes. Increased mitochondrial oxidative capacity may support fatty-acid oxidation and metabolic flexibility; however, changes in mitochondrial content or oxidative-enzyme activity do not necessarily demonstrate improved insulin sensitivity or glycemic control. Indeed, skeletal-muscle glucose uptake is regulated by multiple mechanisms, including insulin-dependent and contraction-mediated glucose transport, GLUT4 availability, and glucose delivery through the microvasculature [90]. In a small, uncontrolled study involving eight individuals with type 2 diabetes, two weeks of low-volume HIIT reduced mean 24-h glucose concentrations and increased markers of skeletal-muscle mitochondrial oxidative capacity, including CS activity and respiratory-chain protein abundance [91]. Although all participants completed the intervention without reported complications, the absence of a control group and direct insulin-sensitivity measurements precludes causal attribution of the glycemic improvements to mitochondrial remodeling or conclusions regarding superiority over MICT.

In individuals with metabolic syndrome, a 16-week randomized pilot study reported greater improvements in maximal oxygen uptake and several cardiometabolic and insulin-signaling outcomes following aerobic interval training compared with MICT matched for prescribed exercise volume [92]. However, the small sample size and assessment of multiple physiological endpoints preclude attributing these benefits exclusively to mitochondrial adaptations. In adults with obesity, eight weeks of HIIT increased skeletal-muscle markers of oxidative metabolism, including β-HAD and COX-IV protein abundance, irrespective of baseline insulin-resistance status [93]. Furthermore, a 12-week comparison of HIIT and MICT in older adults with obesity demonstrated increased TFAM abundance following both interventions, whereas significant within-group increases in TOM20, MFN2, and PARKIN were observed only following HIIT [94]. However, statistically significant between-group differences were not established for these mitochondrial protein markers, and differences in exercise modality and small muscle-biopsy subsamples limit causal interpretation. These findings support the potential clinical relevance of both training approaches but do not establish a universally superior modality across such clinical populations.

A 2025 systematic review and meta-analysis of 394 participants with type 2 diabetes reported improvements in skeletal-muscle markers of oxidative and antioxidant capacity, whereas the pooled effect on mitochondrial content-related markers was not statistically significant (SMD = 0.50, *p* = 0.091) [95]. However, substantial between-study heterogeneity, the predominance of non-randomized designs, and inadequate reporting of antidiabetic medication use limit causal interpretation. These findings underscore the importance of distinguishing mitochondrial content markers from oxidative capacity and directly measured respiratory function. In older adults, HIIT has generally demonstrated favorable feasibility and tolerability within the populations studied, although differences in physical capacity, comorbidities, and intervention protocols, together with inconsistent adverse-event reporting, limit conclusions regarding comparative safety and optimal exercise prescription [96,97]. Accordingly, mitochondrial markers should be interpreted alongside functional outcomes, while exercise prescription should reflect individual capacity, comorbidities, and tolerability; comparative safety and optimal HIIT dosing remain insufficiently established [96,97].

## 6. Conclusions

Both HIIT and MICT can stimulate mitochondrial adaptations in human skeletal muscle through partly overlapping signaling pathways involving AMPK, p38 MAPK, Ca^2+^-dependent signaling, and PGC-1α. When exercise is equated for total work, some molecular responses are comparable at the whole-muscle level, although differences in mitochondrial content and respiratory function can still emerge. Depending on the matching strategy and exercise protocol, HIIT or SIT can elicit mitochondrial adaptations comparable to those observed following continuous training, sometimes with substantially lower accumulated exercise volume or total time commitment. The metabolic stress hypothesis offers a testable explanation for some protocol-specific signaling responses, but its proposed mediators have not been established as sufficient causes of long-term mitochondrial adaptation. Critically, mitochondrial content and respiratory function represent distinct dimensions of adaptation and should be assessed in parallel rather than inferred from a single class of biomarkers. Resolving the contributions of sex, fiber type, mitochondrial dynamics, training status, and metabolic context will be essential for determining how exercise intensity and volume interact to shape mitochondrial adaptation and for developing evidence-based exercise strategies to promote mitochondrial health across the lifespan. Interpretation should additionally consider mitochondrial synthesis and turnover, compartment-specific species limitations, and clinically relevant metabolic outcomes [14,15,22,62,64,67,91,92,95,96,97].

## Figures and Tables

**Table 1:** Selected human comparisons of HIIT & MICT: matching strategy and skeletal-muscle outcomes.

Study	Design	Outcomes	Conclusion
**Bartlett et al. (2012) [12]** *n* = 10 active men Randomized crossover	**HIIT:** 6 × 3 min running at 90% VO_2_max, with recovery at 50%. **MICT:** 50 min running at 70% VO_2_max. **Matched:** Mean intensity, duration and distance.	**Metabolic:** Blood lactate higher after HIIT; glycogen depletion similar. **Molecular:** AMPK/p38 phosphorylation (post-exercise) and PGC-1α mRNA (3 h) increased in both groups without between-group difference.	Different intensity patterns did not yield detectably different measured acute signals.
**Fiorenza et al. (2018) [14]** *n* = 12 trained men Randomized, counterbalanced crossover	**Groups:** Repeated sprints (RS), speed endurance (SE), continuous moderate-intensity exercise (CM). **Matched:** RS and SE were work-matched; CM had higher exercise volume.	**Metabolic:** SE elicited greater muscle lactate & pH changes. **Molecular:** Greater CaMKII & p38 MAPK phosphorylation in SE. PGC-1α and DRP1 mRNA rose more after SE and CM than RS; SE and CM did not significantly differ.	Intensity, metabolic perturbation and volume are not interchangeable explanations.
**MacInnis et al. (2017) [19]** *n* = 10 active men Within-participant, one leg per condition 2 weeks/6 sessions	**HIIT:** 4 × (5 min at 65% Wpeak + 2.5 min at 20% Wpeak). **MICT:** 30 min at 50% Wpeak. **Matched:** Total work and duration.	**Between-condition:** Greater post-training CS activity and mass-specific complex I/I+II respiration with HIIT (interaction effect). No differential change in respiration per unit of CS activity. No group differences in COX IV, MFN2, and NDUFA9 content.	HIIT favored selected markers of mitochondrial amount and mass-specific capacity. Cannot infer superior intrinsic mitochondrial respiration per content.
**Skelly et al. (2023) [32]** *n* = 10 (5 w, 5 m) Within-participant, one leg per condition 4 weeks/12 sessions	**HIIT:** 10 × (3 min at 50% Wpeak, 1 min at 10% Wpeak). **MICT:** 30 min at 50% and 10 min at 10% Wpeak **Matched:** Absolute & mean intensity, work, time.	**Within-condition:** CS activity, NDUFA9 content and subsarcolemmal mitochondrial volume increased after MICT, not HIIT. **Between-condition:** Post-training COX IV content higher after MICT; total mitochondrial volume not significantly changed.	Some evidence favors MICT when work-bout intensity is matched.
**Granata et al. (2016) [33]** *n* = 29 healthy men Parallel groups 4 weeks/12 sessions	**Groups:** SIT (4–10× all-out 30-s bouts), HIIT (4–7× 4-min at 90% Wpeak), and MICT (20–36 min at 65% Wpeak). **Matched:** HIIT–MICT work-matched.	**Within-condition:** PGC-1α & p53 content increased after SIT only. No changes in CS activity, TFAM & MFN2. **Between-condition:** Maximal mitochondrial respiration & PHF20 after SIT greater.	Training mode influenced detected outcomes.

Selected human studies comparing interval and continuous exercise protocols with respect to acute molecular signaling and chronic skeletal-muscle mitochondrial adaptations. Studies differ in exercise modality, matching strategy, intervention duration, and outcome assessment. Acute signaling responses should not be interpreted as direct evidence of subsequent mitochondrial adaptation. The table summarizes selected comparative evidence rather than providing an exhaustive or quantitatively pooled assessment. “Between-condition” = explicitly reported cross-protocol comparison; “within-condition” = pre–post change (not evidence of between-protocol superiority). CS = citrate synthase; COX IV = Cytochrome c oxidase subunit IV, NDUFA9 = NADH: ubiquinone oxidoreductase subunit A9, MFN2 = mitofusin 2, TFAM = Mitochondrial transcription factor A, PHF20 = plant homeodomain finger–containing protein 20, HIIT = high-intensity interval training; MICT = moderate-intensity continuous training; SIT = sprint interval training; Wpeak = peak power output.

## Data Availability

No new data were created or analyzed in this study. Data sharing is not applicable to this article.

## Abbreviations

The following abbreviations are used in this manuscript:

HIIT	High-intensity interval training
MICT	Moderate-intensity continuous training
AMPK	AMP-activated protein kinase
P38 MAPK	p38 mitogen-activated protein kinase
PGC-1α	Peroxisome proliferator-activated receptor gamma coactivator 1-alpha
SIT	Sprint interval training
SIRT1	Sirtuin-1
CS	Citrate Synthase
CaMKII	Ca^2+^/calmodulin-dependent protein kinase II
Nox2	NADPH oxidase 2
NRF	Nuclear respiratory factor
TCA	Tricarboxylic acid cycle
mtDNA	Mitochondrial DNA
MCU	Mitochondrial Calcium Uniporter
OXPHOS	Oxidative phosphorylation
ROS	Reactive Oxygen Species
ND1	NADH Dehydrogenase Subunit 1
Cox2	Cytochrome c Oxidase Subunit 2
TFAM	Transcription Factor A, Mitochondrial
NRF	Nuclear Respiratory Factors
ERR	Estrogen-related receptor
VDAC	Voltage-Dependent Anion Channel
SR	Sarcoplasmic Reticulum
ATF2	Activating Transcription Factor 2
MEF2	Myocyte Enhancer Factor 2
GCN5	General Control Non-derepressible 5
4E-BP1	Eukaryotic Translation Initiation Factor 4E-Binding Protein 1
S6K1	Ribosomal protein S6 kinase
mTOR	Mechanistic target of rapamycin
TOM20	Translocase of the Outer Mitochondrial Membrane 20
YY1	Yin Yang 1
PDK4	Pyruvate Dehydrogenase Kinase 4
FoxO	Forkhead-Box O
PI3K-AKT	Phosphoinositide 3-kinase—Protein Kinase B
LC3	Microtubule-associated protein 1 light chain 3
PINK1	PTEN-induced kinase 1
BNIP3	BCL2-interacting protein 3
NIX/BNIP3L	BCL2/adenovirus E1B 19-kDa-interacting protein 3-like receptor
FUNDC1	FUN14 Domain-Containing Protein 1
